# Age-Related Stereotype Threat and Its Impact on Working Memory in Older Adults: A Systematic Review of the Literature

**DOI:** 10.1093/geroni/igaf122.3880

**Published:** 2025-12-31

**Authors:** Julie Pham, Thomas Ritz, Holly Bowen

**Affiliations:** Southern Methodist University, Dallas, Texas, United States; Southern Methodist University, Dallas, Texas, United States; Southern Methodist University, Dallas, Texas, United States

## Abstract

Stereotype threat related to aging represents a growing area of concern, with evidence suggesting it may impair functions like working memory that are vulnerable to age-related decline. This systematic review examines how age-related stereotype threat impacts working memory in older adults. The current review synthesizes 20 empirical studies (across 22 samples) that used experimental stereotype threat manipulations and assessed working memory outcomes in participants aged 59 and older. Studies were coded by working memory component and function, type of stereotype threat (blatant versus subtle), and sample characteristics. Findings indicate that subtle stereotype cues may impair working memory performance more consistently than blatant threats. Specifically, five of eight samples (62.5%) exposed to subtle stereotype threat showed significant impairments, particularly on tasks involving storage, manipulation, and updating. In contrast, only three of 14 samples (21.4%) exposed to blatant stereotype threat showed significant effects. When analyzing specific working memory components, five of the 13 samples assessing the central executive component showed poorer performance under threat, including two under subtle threat. Of the six samples examining the phonological loop, two found significant effects under subtle threat, as did both studies on visuospatial working memory. One study also reported that subtle threat impaired overall working memory performance. No consistent link was found between participant demographics and stereotype threat effects. Overall, the review suggests that stereotype threat, especially in subtle forms, may compound age-related working memory decline, and highlights the need for more rigorous studies to clarify these effects and guide interventions to support cognitive aging.

